# Altered Daytime Fluctuation Pattern of Plasminogen Activator Inhibitor 1 in Type 2 Diabetes Patients with Coronary Artery Disease: A Strong Association with Persistently Elevated Plasma Insulin, Increased Insulin Resistance, and Abdominal Obesity

**DOI:** 10.1155/2015/390185

**Published:** 2015-05-18

**Authors:** Katarina Lalić, Aleksandra Jotić, Nataša Rajković, Sandra Singh, Ljubica Stošić, Ljiljana Popović, Ljiljana Lukić, Tanja Miličić, Jelena P. Seferović, Marija Maćešić, Jelena Stanarčić, Milorad Čivčić, Iva Kadić, Nebojša M. Lalić

**Affiliations:** ^1^Clinic for Endocrinology, Diabetes and Metabolic Disorders, Clinical Centre of Serbia, Dr. Subotica 13, 11000 Belgrade, Serbia; ^2^Faculty of Medicine, University of Belgrade, Dr. Subotica 8, 11000 Belgrade, Serbia

## Abstract

This study was aimed at investigating daily fluctuation of PAI-1 levels in relation to insulin resistance (IR) and daily profile of plasma insulin and glucose levels in 26 type 2 diabetic (T2D) patients with coronary artery disease (CAD) (group A), 10 T2D patients without CAD (group B), 12 nondiabetics with CAD (group C), and 12 healthy controls (group D). The percentage of PAI-1 decrease was lower in group A versus group B (4.4 ± 2.7 versus 35.0 ± 5.4%; *P* < 0.05) and in C versus D (14.0 ± 5.8 versus 44.7 ± 3.1%; *P* < 0.001). HOMA-IR was higher in group A versus group B (*P* < 0.05) and in C versus D (*P* < 0.01). Simultaneously, AUCs of PAI-1 and insulin were higher in group A versus group B (*P* < 0.05) and in C versus D (*P* < 0.01), while AUC of glucose did not differ between groups. In multiple regression analysis waist-to-hip ratio and AUC of insulin were independent determinants of decrease in PAI-1. The altered diurnal fluctuation of PAI-1, especially in T2D with CAD, might be strongly influenced by a prolonged exposure to hyperinsulinemia in the settings of increased IR and abdominal obesity, facilitating altogether an accelerated atherosclerosis.

## 1. Introduction

Fibrinolysis in blood is mediated by activation of tissue type plasminogen activator (t-PA) whose main role is to convert circulating plasminogen to plasmin resulting in lysis of clots and thrombi. The specific inhibitor of t-PA, plasminogen activator inhibitor 1 (PAI-1), a circulating protein, could attenuate the activity of the fibrinolytic system with consequent thrombus formation [1]. Moreover, it has been clearly shown that overexpression of PAI-1 in the vessel wall predisposes to the development of vulnerable plaques formation and acute coronary syndromes [2]. Previous studies found significantly increased levels of PAI-1 in patients with myocardial infarction (MI) [3], stable or unstable coronary artery diseases (CAD) [4], or even endothelial dysfunction [5]. In addition, increased concentration of PAI-1 in blood and arterial wall was also found in patients with obesity [6, 7], metabolic syndrome [8], and type 2 diabetes [9, 10]. Those results implied that increased expression of PAI-1, possibly induced by insulin resistance and hyperinsulinemia as major metabolic impairments underlying these diseases, could be a factor contributing to premature CAD frequently seen in patients with diabetes [2]. Furthermore, investigations in animal models have shown that infusion of insulin and proinsulin [11], as well as acute hyperglycemia and hyperinsulinemia [12], increased expression of PAI-1. In humans, localized intra-arterial infusions of insulin also induced increase in the concentration of PAI-1 in blood facilitating impaired fibrinolysis [13]. However, clamp studies performed in relatively small number of obese subjects (some of them with diabetes) do not confirm that insulin acutely influenced fibrinolysis. It was speculated that hypofibrinolysis due to increased PAI-1 activity, detected in patients with obesity or T2D, may be linked rather to chronic hyperinsulinism, that is, insulin resistance [14].

It was known for many years that fibrinolytic activity in humans is significantly reduced in the morning mainly due to the highest value of PAI-1 activity at 06:00–08:00 and the nadir in the late afternoon (18:00) [15, 16]. Recently, it was shown that the morning peak of PAI-1 in healthy subjects is caused by internal circadian system independent of sleep/wake cycle and is not induced by behaviors that occur in the morning (altered posture or physical activity) [17]. Investigations done in patients with acute MI have shown that increased PAI-1 together with decreased fibrynolisis in the morning, found in these patients, could be associated with the morning peak of appearance of acute coronary syndrome which was frequently observed [18]. Although some rare previous studies have detected alteration in diurnal fluctuation of PAI-1 in patients with CAD [19], the impairments of PAI-1 circadian variation in T2D, especially in the presence of CAD, remain still unclarified. Therefore, this study was aimed at investigating the daily fluctuation of PAI-1 activity in T2D and nondiabetic patients with previously diagnosed CAD and to examine the relationship of these fluctuations to the changes in plasma insulin, insulin resistance, glucose, and lipid as well as anthropometric parameters, previously suggested to be possible determinants of PAI-1 daily profile in those patients.

## 2. Material and Methods

### 2.1. Study Populations

In this study, we included 60 T2D and nondiabetic patients divided into the groups according to the presence of CAD: 26 T2D patients with documented CAD (group A), 10 T2D without CAD (group B), and 12 nondiabetic patients with CAD (group C). Group D consisted of 12 healthy controls subjects. T2D was previously diagnosed according to the WHO criteria [20]. Patients with diabetes were treated by diet and/or oral agents (metformin and sulfonylurea) and none of them was using thiazolidinediones or insulin. The CAD was diagnosed by cardiologists, based on clinical feature, history of myocardial infarction, and stable angina pectoris or was angiographically verified. Patients with unstable angina pectoris, heart failure, acute myocardial infarction or coronary interventions within the last six months, acute or chronic infections, and malignant diseases were not included. None of the patients was on anticoagulants or corticosteroids at the time of the study. All the participants gave their informed consent for the study which was approved by the Institutional Ethics Committee.

### 2.2. Study Protocol

The investigations were performed in each subject included in the study within the same day. After an interview with questions regarding patient medical history, current medical condition, and medication use, anthropometric measurements were done. Body weight and height were measured with a digital scale and body mass index (BMI) was calculated (kg/m^2^), while the relationship between waists and hip circumference (measured by soft tape) was expressed as waist-to-hip ratio (WHR) and served as marker of abdominal obesity. The blood samples for basal laboratory analysis were drawn in the morning, at 08:00, after 12-hour overnight fasting, in a supine position after 30 minutes of rest, from the antecubital vein. The participants were asked to refrain from any physical activity and drinking coffee or alcohol, while smokers were asked to refrain from smoking during the day when testing was done. In order to analyze daytime fluctuation of PAI-1 activity, blood was collected during the day, in hospital settings with standard hospital meals, before and 2 hours after the main meal (at 08:00, 10:00, 13:00, 15:00, and 18:00) from each subject included in the study. Simultaneously, at the same time points, blood samples were collected for determination of plasma glucose and plasma insulin levels. The differences between PAI-1 activity between morning (08:00) and evening values (18:00) were calculated and expressed as percentage of PAI-1 decrease during the day. Also, area under the curve (AUC) was calculated for PAI-1, insulin, and glucose values.

### 2.3. Laboratory Analysis

All assays were performed using commercially available kits on paired samples. Blood for determination of PAI-1 activity in plasma was collected into 5 mL tubes containing 0.5 mL of sodium citrate. The samples were immediately centrifuged for 10 minutes at 3000 g and 4°C, and then plasma was carefully separated, transferred to small vials, and stored at −80°C until analyzed. The level of PAI-1 activity in plasma was determined by using plasminogen/chromogenic plasmin substrate assay (kit Behring, Germany). The normal range for PAI-1 activity by using this test is 0.3–3.5 U/mL with an interassay CV of 7.7%. Plasma glucose was determined by using glucose oxidase method on Beckman glucose analyzer (Beckman Instruments, Fullerton, USA). Plasma insulin levels were measured by radioimmunoassay with double antibodies kits (INEP, Zemun, Serbia). Insulin resistance was estimated using the homeostasis model assessment (HOMA-IR) and was calculated from fasting plasma insulin and glucose levels according to the formula: (insulin (mU/L) × glucose (mmol/L))/22.5 [21]. Determination of lipid parameters, total cholesterol (chol), HDL-chol, and triglycerides concentrations were analyzed using commercial enzymatic kit (Boehringer Mannheim GmbH Diagnostics), while LDL-chol levels were calculated by using standard Friedewald formula.

### 2.4. Statistical Analysis

The statistical analyses were performed using SPSS software, version 17.0 (SPSS Inc., USA), and data are presented as mean ± SD. Kolmogorov-Smirnov test was used for testing normality of data distribution and variables not normally distributed were log-transformed. The presence of significant linear trends in variable distribution was assessed with analysis of variance (ANOVA) with a post hoc comparison using Bonferroni test. Categorical variables were tested with Kruskal-Wallis test. The total integrated response area under the curve (AUC) for PAI-1, glucose, and insulin was calculated by trapezoid method. The Pearson correlation coefficient or the Spearman's rank correlation was used to test the relationship between the variables. In order to analyze the determinants of PAI-1 fluctuations during the day, stepwise multiple regression analysis was performed with the percentage of PAI-1 decrease as a dependent variable. Differences were defined statistically significant if *P* < 0.05.

## 3. Results

### 3.1. Patients Characteristics

The characteristics of the T2D and nondiabetic patients included in the study are shown in Table 1. In both T2D groups the age and duration of diabetes were similar. Also, nondiabetics and control groups did not differ with respect to age and gender, while both groups with CAD (T2D and nondiabetics) were matched for duration of CAD. All three patients groups had similar prevalence of hypertension which was significantly higher in comparison to control group, while prevalence of smokers was similar in all investigated groups. The investigated T2D patients group (groups A and B) did not differ in HbA1c (Table 2).

### 3.2. Anthropometric Parameters

We did not find significant differences in BMI between the investigated groups, while WHR was significantly higher in group A in comparison to group B (*P* < 0.05), as well as in group C in comparison to group D (*P* < 0.001) (Table 2).

### 3.3. Lipid Parameters

The lipid levels in investigated groups are shown in Table 2. Groups A and B did not differ in fasting lipid levels (total chol, its subfractions, LDL-chol and HDL-chol, and triglycerides), although HDL-chol level was lower in group A, but without statistical significance. Also, we did not find any differences in the total chol, LDL-chol, and triglycerides levels between groups C and D, while HDL-chol levels were significantly lower in group C in comparison to group D (*P* < 0.001).

### 3.4. Insulin Resistance

HOMA-IR was significantly higher in group A in comparison to group B (*P* < 0.05), as well as in group C compared to group D (*P* < 0.01). Moreover, HOMA-IR was significantly higher in group A than in group C (*P* < 0.01) (Table 2).

### 3.5. Basal and Daytime Fluctuation of PAI-1 Activity Levels

We found that the basal PAI-1 activity levels were significantly higher in T2D patients with CAD (group A) compared to T2D patients without CAD (group B) (*P* < 0.01), as well as in nondiabetic patients with CAD (group C) compared to control group (group D) (*P* < 0.05). Moreover, the basal PAI-1 activity levels were significantly higher in group A than in group C (*P* < 0.05) (Table 2).

**Figure 1:**
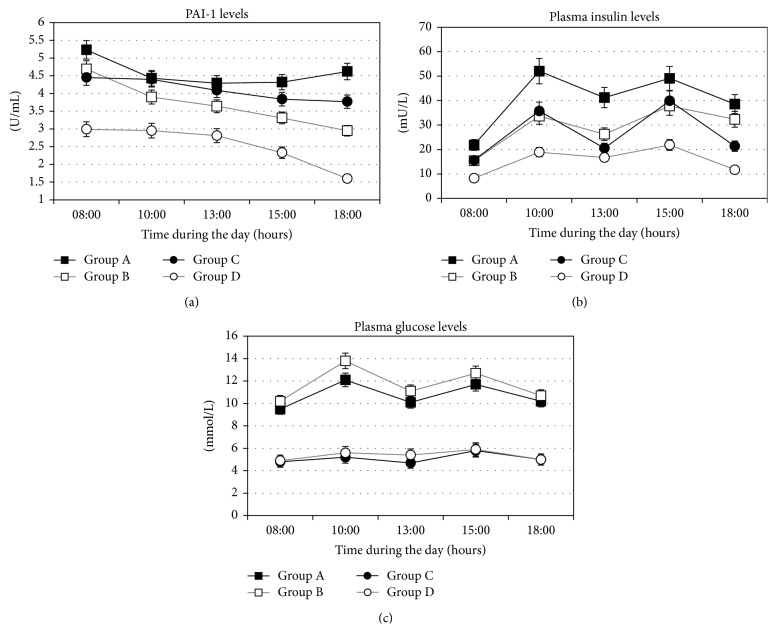
Daily profile of PAI-1 activity (a), plasma insulin (b), and plasma glucose (c) levels in T2D patients with CAD (group A), T2D without CAD (group B), nondiabetics with CAD (group C), and healthy controls (group D). Data are means ± SD.

**Figure 2:**
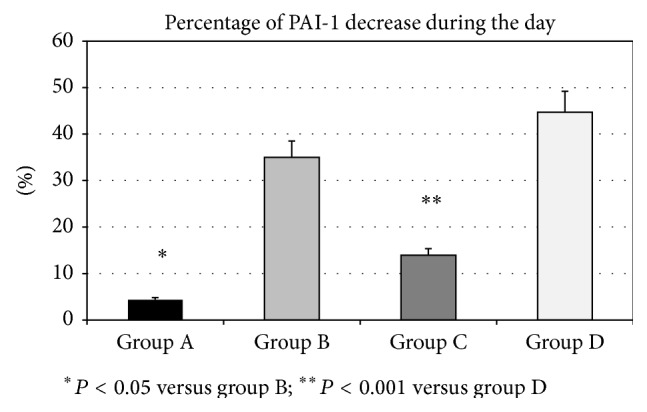
The percentage of PAI-1 decrease during the day, from 08:00 to 18:00, in T2D patients with CAD (group A), T2D without CAD (group B), nondiabetics with CAD (group C), and healthy controls (group D). *P* values indicate the statistical significance of the difference across the groups, as estimated by ANOVA with post hoc Bonferroni test. Data are means ± SD.

**Figure 3:**
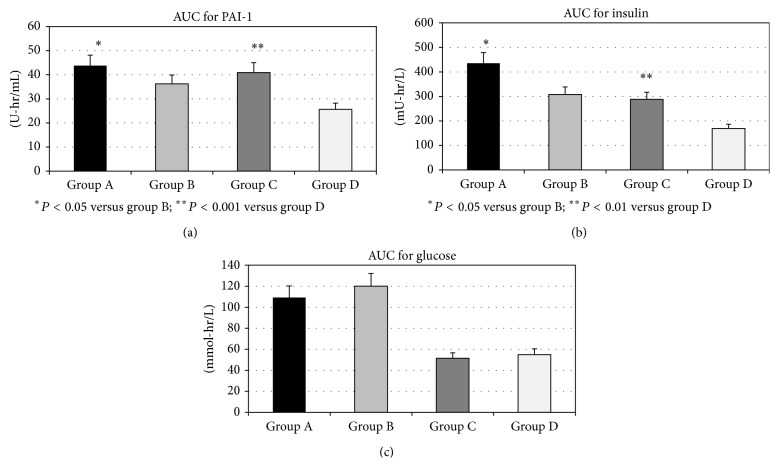
The value of area under the curve (AUC) for daily profile of PAI-1 activity (a), plasma insulin (b), and plasma glucose (c) levels in T2D patients with CAD (group A), T2D without CAD (group B), nondiabetics with CAD (group C), and healthy controls (group D). *P* values indicate the statistical significance of the difference across the groups, as estimated by ANOVA with post hoc Bonferroni test. Data are means ± SD.

The daily profile of PAI-1 levels in all investigated groups was shown in Figure 1(a). In group A, PAI-1 activity levels did not show a significant decrease between 08:00 and 18:00 (5.22 ± 0.27 versus 4.58 ± 0.33 U/mL; *P* = NS). In contrast, in group B, PAI-1 levels decreased during the same interval (4.69 ± 0.38 versus 2.95 ± 0.24 U/mL; *P* < 0.05). Similarly, in group C, we did not find the changes in PAI-1 activity levels during the day (4.45 ± 0.26 versus 3.77 ± 0.24 U/mL; *P* = NS), while they significantly decreased in group D (2.99 ± 0.21 versus 1.60 ± 0.08 U/mL; *P* < 0.001). Moreover, when we compared the percentage of reduction of PAI-1 during the day (08:00 versus 18:00) between the groups, we found that percentage of decrease in PAI-1 was significantly lower in group A than in group B (4.4 ± 2.7 versus 35.0 ± 5.4%; *P* < 0.05), as well as in group C versus group D (14.0 ± 5.8% versus 44.7 ± 3.1%; *P* < 0.001) (Figure 2). Simultaneously, AUC of the PAI-1 activity levels during the day was significantly higher in group A in comparison to group B (43.79 ± 8.16 versus 36.42 ± 7.05 U-hr/mL; *P* < 0.05), as well as in group C in comparison to group D (40.95 ± 7.52 versus 25.67 ± 5.53 U-hr/mL; *P* < 0.001) (Figure 3(a)).

### 3.6. Basal Plasma Insulin Levels and Daily Profile of Plasma Insulin

Basal plasma insulin levels were significantly higher in group A in comparison to group B (*P* < 0.05) and in group C compared to group D (*P* < 0.05), as well as in group A compared to group C (*P* < 0.05) (Table 2). The daily profile of plasma insulin levels in all investigated groups was shown in Figure 1(b). We found that at all investigated time points during the day, plasma insulin levels were higher in group A in comparison to group B, but being statistically significantly different only in the morning (at 8:00 h, *P* < 0.05). However, AUC of insulin levels during the day was significantly higher in group A than in group B (435.56 ± 270.14 versus 308.09 ± 99.71 mU-hr/L; *P* < 0.05). When we compared group C and group D, we also found that plasma insulin levels at all investigated time points were significantly higher in group C. Also, AUC of insulin levels during the day was significantly higher in group C than in group D (288.39 ± 89.01 versus 169.76 ± 17.50 mU-hr/L; *P* < 0.01) (Figure 3(b)).

### 3.7. Fasting Plasma Glucose Levels and Daily Profile of Plasma Glucose

The investigated T2D patients group (groups A and B) did not differ in fasting plasma glucose level. Similarly, we did not found any differences in the glucose level when we compared nondiabetic groups (groups C and D) (Table 2). The daily profile of plasma glucose levels in all investigated groups was shown in Figure 1(c). In contrast to plasma insulin levels, plasma glucose levels were similar at all-time points during the day when group A and group B were compared. Moreover, AUC of plasma glucose levels was similar in groups A and B (109.32 ± 34.66 versus 120.16 ± 31.83 mmol-hr/L; *P* = Ns). Similarly, in groups C and D plasma glucose levels at all investigated time points were without significant difference, as well as AUC of plasma glucose levels (51.38 ± 8.04 versus 54.86 ± 7.08 mmol-hr/L; *P* = NS) (Figure 3(c)).

### 3.8. Analysis of Correlations and Multiple Linear Regression Analysis

In T2D patients, basal PAI-1 activity levels significantly correlated with HOMA-IR (*r* = 0.226; *P* < 0.05), basal plasma insulin levels (*r* = 0.225; *P* < 0.05), and HDL-chol levels (*r* = −0.384; *P* < 0.001), while percentage of PAI-1 decrease during the day significantly correlated only with AUC of insulin levels (*r* = −0.483; *P* < 0.05). However, in nondiabetic patients, basal PAI-1 activity levels significantly correlated with basal plasma insulin levels (*r* = 0.568; *P* < 0.01) and WHR (*r* = 0.228; *P* < 0.05), while correlation with HOMA-IR was on the border of statistical significance (*r* = 0.181; *P* = 0.06). Similarly to T2D patients, in nondiabetics the percentage of PAI-1 decrease during the day significantly correlated only with AUC of insulin levels (*r* = −0.512; *P* < 0.05).

In order to investigate the independent determinants of altered daytime fluctuation pattern of PAI-1 activity levels we performed a stepwise multiple linear regression analysis with the percentage of PAI-1 decrease during the day as dependent variable (Table 3). After collinearity testing was used, in this analysis we included as independent variable HOMA-IR, AUC of plasma insulin, AUC of plasma glucose, LDL-chol, HDL-chol, triglycerides, BMI, and WHR (adjusted for age, gender, and presence of diabetes). We found that only WHR and AUC of plasma insulin were independent determinants of percentage in decrease of PAI-1 during the day, together explaining 26% (*R* ^2^ = 0.261) of the variability of degree of PAI-1 activity fluctuation during the day.

## 4. Discussion

The results from this study have shown that increased basal levels and impaired circadian variations of PAI-1, with protracted high PAI-1 levels during the day, are associated with CAD in patients with T2D as well as in nondiabetics. Moreover, the PAI-1 levels in T2D patients were higher compared to those in nondiabetics, both when basal levels or daytime variations were concerned.

In the past two decades, a number of studies have demonstrated elevated basal levels of PAI-1 in patients with CAD, reflecting impaired fibrinolysis [22–25]. However, the exact mechanisms by which a reduced fibrinolysis may lead to the appearance CAD in T2D are not yet fully understood [26, 27].

The results of testing of PAI-1 in this study are consistent with the above data. We found that the basal level of PAI-1 is significantly higher in patients with T2D and CAD compared with diabetic patients without CAD and in both groups of diabetic patients is significantly higher than in the group of healthy individuals. Also, in nondiabetics, our findings were similar with basal levels of PAI-1 in patients with CAD being significantly higher compared with the group of healthy subjects. Moreover, increased PAI-1 observed in T2D with CAD was significantly higher than in nondiabetics with CAD.

It has been previously shown that the secretion of PAI-1 in healthy individuals shows diurnal variation and that the level of PAI-1 secretion has a circadian rhythm with the highest values in the morning and the lowest values in the afternoon [28]. It has also been shown that in patients with elevated levels of PAI-1 (nondiabetics, persons in a state of infection, and pregnancy) there might be alterations in the daily variations in the level of PAI-1 [16]. On the other hand, some previous data from large studies signify that the highest incidence of acute MI found in patients with CAD is in the early morning [29], which is also the period of the peak of PAI-1. Later, it was shown that under condition of elevated morning PAI-1, coronary atherosclerotic plaque could become vulnerable and prone to rupture, thereby precipitating acute coronary syndromes [2]. In contrast, recently it was shown that presence of diabetes completely abolished the morning predominance of MI and that a circadian pattern of MI onset could not be verified [30]. However, it is not known whether these influence of diabetes is solely due to the disappearance of daily variations of PAI-1 in patients with diabetes or that changes in insulin levels and insulin resistance might also be important contributors.

In our study, major new findings are that loss of circadian variation of PAI-1 was more significantly expressed in T2D patients with CAD compared to nondiabetics with CAD. To the best of our knowledge, this difference has not been demonstrated in the available literature. Some previous studies have shown minor impairment in diurnal variation of PAI-1, but only in male nondiabetic patients with CAD [19]. The authors found morning increase in PAI-1 in those patients, with 48% decrease in PAI-1 until evening. We also found increase in PAI-1 levels in the morning in nondiabetic patients (but both males and females) with CAD, although the evening decrease of PAI-1 in nondiabetics was remarkably lower (14%). In addition, in diabetic patients with CAD in our study, percentage of PAI-1 decrease during the day was the lowest (only 4.4%), showing an almost flat daytime curve of PAI-1 in these patients. This strongly suggested that significantly disturbed diurnal rhythm of PAI-1 secretion in these patients is related to appearance of CAD. The other similar investigation in patients with unstable and stable angina (some of them were diabetics) has shown significantly higher levels of PAI-1 during the whole day (measured at three time points: morning, early afternoon, and late evening) in both patients groups compared to healthy controls [31]. However, in our study, patients with diabetes were separated from nondiabetics. We also found higher whole daily levels of PAI-1 (expressed as AUC of PAI-1), but in both diabetic and nondiabetic groups with CAD (group A and group C) in comparison to corresponding groups without CAD, while in patients with CAD the daytime PAI-1 levels were higher in T2D than in controls.

Simultaneously, we have demonstrated significantly higher levels of insulin during the day in the patients with CAD, more expressed in T2D than in nondiabetics, which correlated with impairment in diurnal variation of PAI-1. These findings imply that exposure to hyperinsulinemia throughout the day could strongly contribute to the accelerated atherosclerosis seen in patient with diabetes. The relationship between insulin and PAI-1 has been investigated extensively in previous years. More than 20 years ago, the effects of insulin on PAI-1 have been studied in vitro, in cell cultures of endothelial cells [32] and the hepatocyte cell lines [33] that synthesize PAI-1. It has been shown that the addition of insulin to the culture of hepatocytes, in concentration which exists in the portal vein after the meal, leads to a double increase in the production of PAI-1 in these cells, without modifying other hepatocyte products [34]. In addition, insulin, but also triglycerides and in some studies hyperglycemia, could increase the expression of PAI-1 by human arterial segments in vitro, both in normal vasculature and within atherosclerotic plaques [35]. These results from in vitro studies have demonstrated that this combination of metabolic impairments underlying T2D, usually associated with insulin resistance (see below), directly influence hypofibrinolysis and facilitate atherosclerosis in those patients [2]. In contrast, in vivo, these direct effects of insulin infusion on PAI-1 have not been observed so far, but investigations were done only in healthy subjects [36, 37], although studies in animal models suggested that infusion of insulin and proinsulin [11] increased expression of PAI-1. Results from our study are suggesting that higher insulin levels during the whole day could be related to observed impairments in fluctuation of PAI-1 during the day in both diabetic and nondiabetic patients with CAD, being more expressed in the T2D patients.

Previous study also suggested that insulin resistance, measured using different methodology, mostly HOMA-IR, is associated with CAD in patients with T2D [38]. One of the rare studies in which insulin resistance was directly measured using the clamp techniques has clearly shown that in diabetic patients insulin resistance is associated with vascular damage and impaired fibrinolysis, independent of obesity and poor glycemic control [39]. In line with these data are the findings in our study that T2D patients with CAD had more pronounced insulin resistance, measured by HOMA-IR, than diabetic patients without CAD. In addition, observed elevated basal PAI-1 level significantly correlated with HOMA-IR and plasma insulin levels, predominantly in the T2D patients.

Interestingly, it seems according to our results that hyperglycemia in the T2D patients is not related to diminished daily fluctuation in PAI-1, as it was already shown in clinical studies by other authors [40]. Also, we did not find differences in total LDL-chol and triglycerides between T2D patients with and without CAD, as well as when we compared nondiabetics with CAD and healthy controls, while HDL-chol levels were lower in both groups with CAD. Moreover, increased basal PAI-1 level significantly correlated with HDL-chol, but only in T2D patients. In accordance with our results, Mertens et al. demonstrated that PAI-1 activity was significantly related to HDL-chol in T2D patients free of CAD [41]. They also pointed out a strong relationship between PAI-1 and LDL peak particle diameter but not with LDL cholesterol levels.

Although the triglycerides did not differ in fasting conditions between groups with and without CAD in our study, the triglyceride levels during the day might be a factor potentially influencing the diurnal fluctuation of PAI-1, although we were not able to measure them in this study. In this context, some rare studies have previously shown that postprandial hypertriglyceridemia is associated with inflammatory and procoagulant state (including increase in PAI-1), but after high-fat meal and only in hypertensive patients [42].

In our study, we found that patients with CAD, both in T2D and nondiabetic, had higher WHR than those without CAD and healthy controls. Simultaneously, there were no differences in BMI between the groups, implying that fat distribution is more important than overall obesity as a risk for CAD. This is in accordance with previous studies showing that abdominal obesity is well-documented risk factors for cardiovascular disease and T2D [43]. Also, abdominal obesity has been found to be associated with insulin resistance and increased PAI-1 concentrations [7, 44, 45]. Moreover, it was previously clearly shown that abdominal visceral fat expressed 5-fold more PAI-1 than subcutaneous tissue [46]. In accordance with those data, the results from multiple regression analysis in our study have shown that impairments in daily fluctuation of PAI-1 were significantly and independently influenced only by WHR, as a marker of abdominal obesity, and AUC of insulin. These findings suggest that prolonged and chronic hyperinsulinemia, in the settings of abdominal obesity and insulin resistance, exhibits its atherogenic role through impairment of daily circadian rhythm of PAI-1.

## 5. Conclusion

In our results, we have demonstrated the alterations in the diurnal fluctuation pattern of PAI-1 activity, remaining persistently high instead of the afternoon decline, both in T2D patients and nondiabetics with CAD, which is more expressed in T2D. In addition, we have shown that these alterations are strongly influenced by a prolonged daily exposure to elevated plasma insulin levels, especially in T2D with CAD. The influence is suggested to be determined by coexistent insulin resistance and abdominal obesity, which altogether contribute to accelerated atherosclerosis in these patients.

## Figures and Tables

**Table 1:** Patient characteristics.

Variables	Group A	Group B	Group C	Group D
	T2D+ CAD+	T2D+ CAD−	T2D− CAD+	Controls
*n*	26	10	12	12
Gender (m/f)	18/8	3/7	9/3	5/7
Age (years)	56.9 ± 9.2^∗^	54.5 ± 7.2	55.9 ± 11.0	53.9 ± 11.0
Duration of diabetes (years)	9.8 ± 4.2	9.6 ± 3.6	/	/
Duration of CAD (years)	6.9 ± 4.5	/	6.2 ± 3.0	/
Hypertension (*n*, %)	18 (69.2)	7 (70.0)	9 (75.0)	4 (33.3)^a^
Smokers (*n*, %)	13 (50.0)	4 (40.0)	6 (50.0)	7 (58.0)

^∗^Data are expressed as mean ± SD; T2D: type 2 diabetes; CAD: coronary artery disease.

^a^ *P* < 0.05 versus groups A, B, and C.

**Table 2:** Metabolic and anthropometric parameters.

Variables	Group A	Group B	Group C	Group D	*P*-trend ANOVA
	T2D+ CAD+	T2D+ CAD−	T2D− CAD +	Controls	
Fasting glucose (mmol/L)	8.5 ± 2.5^∗^	9.2 ± 2.3	5.2 ± 0.6	5.0 ± 0.9	<0.001
HbA1c (%)	7.6 ± 1.2	8.2 ± 1.5	5.3 ± 0.6	5.1 ± 0.6	<0.001
Fasting insulin (mU/L)	22.97 ± 2.99	13.29 ± 1.72^a^	13.19 ± 1.30	10.12 ± 3.88^b^	0.018
HOMA-IR	9.3 ± 1.9	5.6 ± 1.0^a^	3.1 ± 1.2	2.2 ± 0.8^b^	0.043
Total chol (mmol/L)	6.6 ± 1.1	6.6 ± 0.9	7.1 ± 1.2	6.2 ± 0.9	0.262
HDL-chol (mmol/L)	1.15 ± 0.38	1.39 ± 0.50	1.25 ± 0.37	1.56 ± 0.30^b^	0.023
LDL-chol (mmol/L)	4.3 ± 1.0	3.8 ± 1.0	4.8 ± 0.9	3.9 ± 0.9	0.127
Triglycerides (mmol/L)	2.26 ± 1.12	2.97 ± 1.20	1.91 ± 0.81	1.52 ± 0.52	0.010
PAI-1 (U/mL)	5.58 ± 1.48	3.83 ± 1.03^a^	4.34 ± 0.82	2.68 ± 0.63^b^	<0.001
BMI (kg/m^2^)	27.5 ± 2.9	28.3 ± 4.7	27.2 ± 3.2	25.6 ± 3.6	0.116
WHR	0.97 ± 0.07	0.94 ± 0.08^a^	0.95 ± 0.06	0.83 ± 0.08^b^	<0.001

^∗^Data are expressed as mean ± SD; T2D: type 2 diabetes; CAD: coronary artery disease; HOMA-IR: homeostasis model for insulin resistance; chol: cholesterol; PAI-1: plasminogen activator inhibitor 1; BMI: body mass index; WHR: waist-to-hip ratio.

^a^Group A versus group B: *P* < 0.05 for fasting insulin, HOMA-IR and WHR; *P* < 0.01 for PAI-1 (post hoc comparisons using Bonferroni test).

^b^Group C versus group D: *P* < 0.05 for fasting insulin, HOMA-IR, HDL-chol and PAI-1; *P* < 0.001 for WHR (post hoc comparisons using Bonferroni test).

**Table 3:** The variable independently related to altered daytime fluctuation pattern of PAI-1 in multiple stepwise linear regression analyses.

	*B* (95% CI)	*β*	*R* ^2^	Adjusted *R* ^2^	*P* value
(1) WHR	−89.05 (−162.54 to −15.56)	−0.357	0.167	0.145	0.019
(2) AUC of insulin	−0.04 (−0.07 to −0.02)	−0.310	0.261	0.220	0.040

Dependent variable in the model: percentage of PAI-1 decrease during the day.

Independent variable in the model: HOMA-IR, AUC of plasma insulin, AUC of plasma glucose, LDL-chol, HDL-chol, triglycerides, BMI, and WHR (adjusted for age, gender, and presence of diabetes).
